# TSCC: Two-Stage Combinatorial Clustering for virtual screening using protein-ligand interactions and physicochemical features

**DOI:** 10.1186/1471-2164-11-S4-S26

**Published:** 2010-12-02

**Authors:** Daniel L Clinciu, Yen-Fu Chen, Cheng-Neng Ko, Chi-Chun Lo, Jinn-Moon Yang

**Affiliations:** 1 Institute of Bioinformatics and Systems Biology, National Chiao Tung University, 75 Bo Ai Street, Hsinchu, 30050, Taiwan; 2 Department of Biological Science and Technology, National Chiao Tung University, 75 Bo Ai Street, Hsinchu, 30050, Taiwan; 3 Core Facility for Structural Bioinformatics, National Chiao Tung University, 75 Bo Ai Street, Hsinchu, 30050, Taiwan; 4 Institute of Information Management, National Chiao Tung University, 1001 University Road, Hsinchu, 30010, Taiwan

## Abstract

**Background:**

The increasing numbers of 3D compounds and protein complexes stored in databases contribute greatly to current advances in biotechnology, being employed in several pharmaceutical and industrial applications. However, screening and retrieving appropriate candidates as well as handling false positives presents a challenge for all post-screening analysis methods employed in retrieving therapeutic and industrial targets.

**Results:**

Using the TSCC method, virtually screened compounds were clustered based on their protein-ligand interactions, followed by structure clustering employing physicochemical features, to retrieve the final compounds. Based on the protein-ligand interaction profile (first stage), docked compounds can be clustered into groups with distinct binding interactions. Structure clustering (second stage) grouped similar compounds obtained from the first stage into clusters of similar structures; the lowest energy compound from each cluster being selected as a final candidate.

**Conclusion:**

By representing interactions at the atomic-level and including measures of interaction strength, better descriptions of protein-ligand interactions and a more specific analysis of virtual screening was achieved. The two-stage clustering approach enhanced our post-screening analysis resulting in accurate performances in clustering, mining and visualizing compound candidates, thus, improving virtual screening enrichment.

## Background

Continuous advancements in high-throughput X-ray crystallography and genomics [1,2] account for numerous available three-dimensional (3D) structures, enabling the development of new potential therapeutic and industrial targets. However, prospective ligands and proteins need to be screened in order to downsize groups [3-7] and select suitable candidates for post-screening analysis. Clustering methods based on structural similarity which are employed in post-screening analysis generally improve the scoring function performance. In developing methods for 3D compound retrieval, a detailed understanding of intermolecular interactions between proteins and their ligands is critical to structure-based inhibitor design. Various post-screening analysis methods clustering and [8-13] employ the root mean square deviation (RMSD), protein-ligand interactions and computation and comparison platforms for measuring distances. Since the above methods as well as TSCC encounter challenges of specific selectivity and false positives, we aim to provide advantages of our cluster analysis method to to enrich accuracy and effectively mine candidates for bioassay.

One of the above methods, a post-screening analysis for visualizing protein-ligand interaction (VISCANA) which analyzes the receptor and ligand pattern of interaction on the basis of quantum theory is an approach proposed by Amari *et al*[12]. VISCANA applies the *ab initio* fragment molecular orbital (FMO) method [14] to represent the interaction between a protein and its ligand. The FMO method has an advantage of describing the charge-transfer between a receptor and a ligand in comparison to a conventional force field method using fixed atomic charges. However, it lacks sufficient descriptions of van der Waals forces and hydrogen bond interactions which play an important role in receptor-ligand binding and this may account for additional false positives.

Another method is NIPALSTREE, an approach by Bocker *et al*[13] for clustering large datasets in high dimensional space. It uses the first Principal Component which employs NIPALS (non-linear iterative least squares) where the data set is split at point i or j (determined points where two neighbors exceed a predefined distance threshold T). Calibur [7] is a new tool designed for clustering very large numbers of protein decoys in ab initio protein structure prediction. Since various post-screening analysis methods deal with increasing number of decoys, it can be a useful tool as it can perform the clustering in one third of the time required if its strategies are not used.

Our goal is to develop a cluster analysis for protein-ligand complexes using protein-ligand molecular interactions. We employed the empirical energy function from GEMDOCK [15] and the basic premise of SIFt [16] to encode additional interaction-specific information into the real number strings, hydrogen bonds, van der Waal and electrostatic forces. By representing interactions at the atomic-level as opposed to the residue level and including measures of interactions strength, protein-ligand interactions can be described better and a more precise analysis of virtual screening can be obtained.

TSCC is accomplished by the joining of two clustering stages; one of protein-ligand interactions (e.g. hydrogen bonds, electrostatic interactions, and van der Waals forces) with another of physicochemical features performed on compounds selected through the first stage of clustering. We employed our docking tool, GEMDOCK, to generate protein-ligand interactions and used the Accelrys Cerius QSAR module for obtaining physicochemical features of complexes. Based on normalized feature profiles, hierarchical and K-mean [17] clustering methods were used to cluster compound candidates. Since clustering based upon similarity requires a quantitative measure (descriptor) of the similarity between two molecules, 2D and 3D methods were used to generate a descriptor such as the atom pair descriptor (i.e. compound topological similarity) [18].

A cluster analysis for selecting candidates from a large number of database compounds requires prior screening techniques which must employ docking and screening tools. To handle the vast results from virtual screening and use more specific information for protein-ligand binding, we utilize the empirical energy function from GEMDOCK specifically optimized for virtual screening. GEMDOCK used piecewise linear potential (PLP) that is a simple scoring function and is comparable to some scoring functions for estimating binding affinities [19-21]. Our previous works showed that GEMDOCK was compared to some docking methods on the 100 protein-ligand complexes and two virtual screening targets [4,22]. In addition, GEMDOCK has been successfully applied to identify inhibitors and binding sites for some targets [23-27]. Here, we currently utilized the PLP of GEMDOCK to generate the protein-ligand interaction profiles.

To demonstrate the efficiency of our method we successfully applied its combinatorial two-stage concept on five common targets by constructing two compound sets to screen against each target protein. The first compound set, a verifying dataset, was used to determine if the protein-ligand interaction descriptor is suitable for identifying compounds with similar binding modes. The second compound set, a testing dataset, was used to evaluate the database enrichment potential and the property of compounds in the same cluster by docking a diverse set of compounds spiked with known inhibitors into the same target protein as shown below.

## Methods

### The Two-Stage Combinatorial Clustering (TSCC) methodology

**Figure 1:**
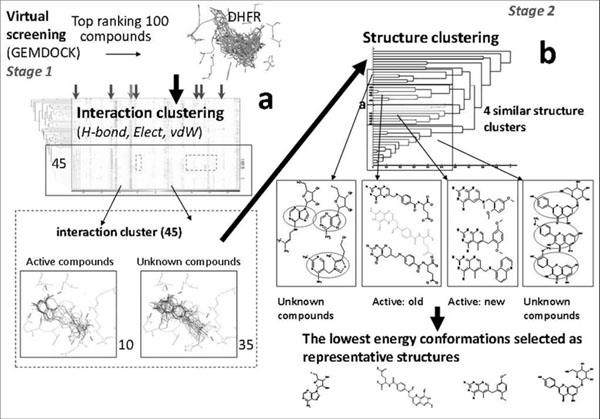
**Overall process of the Two-Stage Combinatorial Cluster Analysis**. (a) First stage clustering using protein-ligand interactions generated via GEMDOCK. (b) Second stage clustering of first stage results done using physicochemical features.

**Figure 2:**
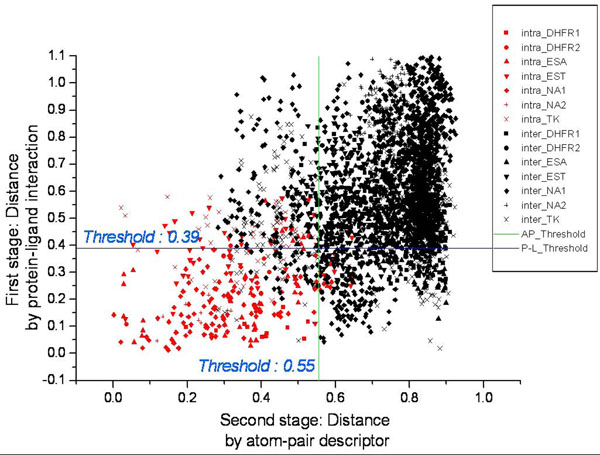
**Designing a reference threshold of P-L interaction and atom-pair descriptors**. The complementation between atom-pair descriptor and the protein-ligand interaction descriptor is also show in this figure. The distance threshold of atom-pair descriptor was 0.55 (tanimoto coefficient). The threshold of distance of protein-ligand interaction descriptor was 0.39 (correlation coefficient).

The overview of our method is shown in **Figure**1. We first calculated the atom-basedprotein-ligand interactions by converting every docked pose into a one dimensional real number string in order to visualize and analyze large data obtained from virtual screening using Yang *et al*[22]. Due to protein-ligand interactions representation, we were able to evaluate the distance of binding modes between two docked poses and to carry out hierarchical clustering analysis. Compounds with a similar binding mode were visualized and grouped into clusters [28]. In our structure based clustering section, each structure was represented by a one dimension atom-pair descriptor, an approach proposed by Carhart *et al*[18]. After analyzing the distance between active and non-active compounds, a reference threshold was decided for demarcating similar compounds (Fig.2).

We generated two sets of structure-based virtual screening results: 1) to verify if the protein-ligand interaction descriptor is suitable for identifying compounds with similar binding mode and 2) to evaluate the database enrichment potential and the property of compounds in the same cluster by docking a diverse set of compounds spiked with known inhibitors into the same target protein.

### Preparation of target protein and compound databases

We tested the virtual screening results against the five target proteins: 1) herpes simplex virus type 1 thymidine kinase (TK) [29] PDB identification (ID): lkim, 2) human estrogen receptor alpha (ER*α*) [22,30] PDB ID: 3ert, 3) human estrogen receptor alpha (ER*α*) PDB ID: lgwr, 4) human dihydrofolate reductase (hDHFR) [31,32] PDB ID: lhfr, and 5) tern n9 influenza virus neuraminidase (NA) [33,34] PDB ID: lmwe.

The ligand binding site was defined as a collection of amino acids using a cutoff radius of 10Å from each atom on the bound ligand, since most studies in lead discovery use a cutoff radius between 8 to 12 Å. Structure files were stored as a PDB format for GEMDOCK input and analysis.

### Compound databases

We constructed two compound sets for screening against each target protein. The compound sets for NA of influenza virus were derived from the Comprehensive Medical Chemistry database (CMC) and only those with molecular weights between 200 and 800 were chosen. All active compounds (61 total) were listed as the following: 1) TK: 10, 2) ER*α* antagonists: 11, 3) ER*α* agonists: 10, 4) hDHFR: 10, and 5) NA: 20. The two crystal structures of human estrogen receptors alpha have been intensively studied for their different functions (agonist 1GWR promotes coactivator binding while antagonist 3ERT blocks it) and ability to bind on the same site of the protein. The agonists play an important role in regulation of gene expression and prevention of osteoporosis while the antagonists have been used as treatment of hormone-dependent breast cancer [22,30].

The tested dataset contained 990 randomly selected compounds combined with known active compounds for each target protein using a method from Bissantz *et al*[35]. The 990 compounds is a small scale public set of compounds used by various studies to test methods of lead discovery. All compound structures were converted to mol formats and their hydrogen atoms removed using CORINA3.0 for GEMDOCK input and VS. The active compound set of each target protein, target proteins, and 990 random compounds are available on the Web at http://gemdock.life.nctu.edu.tw/dock/download.php.

### Preparation of virtual screening results for cluster analysis

GEMDOCK was substantially modified, in preparation for docked poses and to predict the binding affinity for each compound in the dataset *via* two key functions: 1) The searching algorithm and 2) The scoring function which is based on an empirical energy function (it consists of a simple empirical binding score and a pharmacophore-based score with all details of the scoring function found in Additional File 1).

### Testing and Verifying Datasets

The lowest energy conformation was retained for generating the representative docked pose of each compound.

### Generation of Descriptors (Protein-Ligand interaction descriptors)

We converted 3D docked poses into a one dimension real number string by calculating the energy between each atom present on protein and ligand. The interaction energy of each atom *j* on a protein is defined as:(1)

Where  is the distance between atoms *i* and *j* with interaction type *B_ij_* formed by pair-wise heavy atoms between ligands and proteins, *B_ij_* is either a hydrogen bond or a steric state. These two potentials are calculated by the same function, although from different parameters; *V_1_, . . .*, *V_6_. q_i_* and *q_j_* are the formal charges and 332.0 is a factor that converts the electrostatic energy into kilocalories per mole. The *lig* and *pro* denote the number of heavy atoms on the ligand.  is a simple atomic pair-wise potential function.

### Atom pair descriptors

Atom-pair descriptors are 2D topological descriptors counting the distance between twoatoms as the shortest path of bonds [18]. The procedure for preparing atom pair descriptors:

1) Structure files in mol format

2) Remove hydrogen atoms

3) Convert to mol2 format via CORINA3.0

4) Calculate atom pair descriptors via AP generator (distance bins: 15)

5) Store in binary coding form.

A total of 825 (55 x 15) atom pair descriptors were generated for each molecular structure by removing all columns with zero values.

### Reference Threshold for Protein-Ligand Interaction and Atom-Pair Descriptor

To design a reference threshold of protein-ligand interaction, a verifying dataset was used in establishing a reference threshold of distance by determining a maximum discrimination between similar and non-similar binding modes. The equation is as follows:(2)

Where *t* is the reference threshold, *C_intra-d<t_* is the number of intra active compound pairs with the distance < threshold and *C_inter_* is the number of compound pairs between active and non-active compounds.

### The Cluster Analysis Method

First, we used a protein-ligand interaction descriptor for clustering compounds with similar binding modes and applied the correlation coefficients as similarity measurements. The following formula was used:(3)

where  is the correlation distance between docked pose *X* and *Y*. *S_x_* is the standard deviation of *X*. *X_i_* is the *i*th value of *X*. *n* is the number of descriptors. We applied the standard UPGMA clustering method for calculating the distance between two clusters while constructing the dendrogram. The formula is defined as:(4)

The reference threshold was calculated from the verifying dataset using equation (2) to determine the number of clusters.

Second, we applied the AP descriptor for clustering compounds within each clustering stage and applied the tanimoto coefficients as similarity measurements. Formula is as follows:(5)

where  is the tanimoto distance between *X* and *Y. |X⋂Y|* is the number of ON bits common in both *X* and *Y*, and the *|X⋃Y|* is the number of ON bits present in either *X* or *Y*. This equation is similar to equation (4);  by . The dendrogram graph was plotted for visualizing the binding mode of multi docked poses by the protein-ligand interactions.

## Results

### Molecular Recognition

#### Thymidine kinase

Choosing the crystal coordinates of TK (Fig. S1 in Additional File 2) in complex with its natural substrate (deoxythymidine) was reasonable since the active site can accommodate a broad variety of ligands. The average RMSD of all ten docked poses was 1.39 Å. (Table 1)

**Table 1:** The RMSD values between docked poses and crystal ligands of 48 compounds for the five targets

TK (lkim)		ER (3ert, lgwr)		DHFR (lhfr)		NA(lmwe)	
Complex	RMSD	Complex	RMSD	Complex	RMSD	Complex	RMSD
name	(Å)	name	(Å)	name	(Å)	name	(Å)

*le2k.TMC*	0.69	*1err.RAL^a^*	1.27	*lboz.PRD*	1.13	*1l7f_ BCZ*	0.88
*le2m.HPT*	0.51	*3ert.OHT^a^*	0.71	*1dlrMXA*	0.62	*lnnc_GNA*	0.75
*le2n.RCA*	1.34	*1hj1.AOE^a^*	3.13	*1dls.MTX*	1.53	*2qwf_G20*	0.60
*le2p.CCV*	0.67	*1uom.PTI^a^*	0.81	*1drf.FOL*	1.24	*1bji_G21*	0.81
			
*1ki2.GA2*	3.04	*1gwr.EST^b^*	0.71	*1hfr.MOT*	0.51	*1f8b_DAN*	0.64
*lki3.PE2*	3.21	*112i.ETC^b^*	0.52	*1kms.LIH*	1.36	*1f8c_4AM*	0.46
*1ki6.AHU*	0.37	*1qkm.GEN^b^*	2.92	*1kmv.LII*	0.83	*1f8d_9AM*	0.59
*1ki7.ID2*	0.49	*3erd.DES^b^*	1.32	*1mvs.DTM*	0.75	*1f8e_49A*	0.60
			
*lkim.THM*	0.41			*1ohj.COP*	1.27	*1ina_ST6*	0.79
*2ki5.AC2*	3.14			*2dhf.DZF*	1.12	*ling_ST5*	1.03
					
						*1inw_AXP*	0.93
						*1inx_EQP*	0.92
						*1ivc_ST2*	2.09
						*1ivd_ST1*	1.02
						*1ive_ST3*	1.03
						*Imwe_SIA*	0.52
						*lxoe_ABX*	1.33
						*lxog_ABW*	2.42
						*2qwg_G28*	0.80
						*2qwh_G39*	0.74

*Average*	1.39	*Average*	1.42	*Average*	1.03	*Average*	0.95

^a^Four antagonists docked into the target protein (3ert)

^b^Four agonists docked into the target protein (1gwr)

#### Estrogen receptor α

The target protein structures of ERα (Figs. S2 and S3 in Additional Files 3 and 4) were obtained from PDB, whereas antagonists and agonists were derived from previous works. We docked four antagonists into the target protein (3ert) and four agonists into another one (1gwr), and concluded their results based on RMSD in the heavy atoms ligand between the docked pose and the crystal structure. The average RMSD of docked antagonists and agonists was 1.42 Å. The RMSD values of 1hj1.AOE and 1qkm.GEN were larger than 2.0 Å because the native proteins were crystal structures of ER α-ligand complexes. (Table 1)

#### Human dihydrofolate reductase

To evaluate the docking accuracy of GEMDOCK, we docked 10 known active compounds (Fig. S4 in Additional File 5) into the target protein and compared the RMSD values between the docked pose and the bound ligand in crystal structure. The average RMSD of all ten docked active compounds was 1.03 Å, substantially lower than 2 Å, which means GEMDOCK computations were within the range of accepted accurate values.

#### Neuraminidase

The 20 known active compounds (Fig. S5 in Additional File 6 were docked into the target protein and an average RMSD of 0.95 Å was obtained for all docked poses. (Table 1)

### Significance of protein-ligand interaction descriptor on the verifying dataset

#### Significance of known compounds in the five classes

the results are listed in Table 2 using T-scores as the standard two sample *t*-test statistics (Additional File 1). Using equation 2, the maximum discrimination was determined (Fig. 2) with 88.89% accuracy in distinguishing between similar and non-similar binding modes.

**Table 2:** T-test of distance between intra-cluster and inter-cluster compound binding modes generated by converting the docked pose into protein-ligand interaction profile (α=0.01)

Target protein	H_0_	Average distance of intra-cluster compounds (**Å**)	Average distance of inter-cluster compounds (**Å**)	Std^a^ of distance of intra-cluster compounds	Std^a^ of distance of inter-cluster compounds	*p*-value
DHFR	Reject	0.21	0.50	0.09	0.13	1.71E-58
ESA	Reject	0.25	0.42	0.13	0.12	7.04E-20
EST	Reject	0.31	0.48	0.09	0.12	7.94E-39
NA	Reject	0.17	0.73	0.07	0.20	0.00E+00
TK	Reject	0.19	0.47	0.08	0.15	3.89E-64

^a^ Standard Deviation

#### Significance of similar compounds

For the purpose of post-analysis, we tested similar compounds’ docking behavior (pose, interaction) on a protein receptor. There are five classes of similar compounds on each target protein. We tested to see whether the mean distance between similar compounds represented by protein-ligand interactions is different than the mean distance between non-similar compounds (*t*-test results listed in Table 3).

**Table 3:** T-test of distance between intra-cluster and inter-cluster compound structures gener-ated by atom-pair representation (α=0.01)

Target protein	H_0_	Average distance of intra-cluster compounds (**Å**)	Average distance of inter-cluster compounds (**Å**)	Std^a^ of distance of intra-cluster compounds	Std^a^ of distance of inter-cluster compounds	*p*-value
DHFR	Reject	0.42	0.63	0.15	0.12	5.84E-23
ESA	Reject	0.24	0.66	0.11	0.14	4.60E-65
EST	Reject	0.27	0.63	0.14	0.14	2.85E-56
NA	Reject	0.32	0.65	0.18	0.17	1.75E-131
TK	Reject	0.22	0.63	0.09	0.19	2.11E-93

^a^ Standard deviation

#### Significance of an atom pair descriptor

Similar structures were defined as active compounds and non-similar structures were defined as non-active compounds (t-test results, Table 4). Active compounds of hDHFR and NA were divided into two classes because of their diverse compound structures (Figures S2 and S3 - Additional Files 3 and 4). The maximum discrimination between similar and non-similar structures was determined by distinguishing between similar and non-similar structures with 91.45% accuracy.

**Table 4:** T-test of distance between intra-cluster and inter-cluster compounds on each target protein. Descriptor was generated by converting the docked pose into protein-ligand in-teraction profile (α=0.01)

Target protein	Compound class	H_0_	Average distance of intra-cluster compounds (**Å**)	Average distance of inter-cluster compounds (**Å**)	Std^a^ of distance of intra-cluster compounds	Std^a^ of distance of inter-cluster compounds	*p*-value
	DHFR	Reject	0.21	0.50	0.09	0.13	1.71E-58
	ESA	Reject	0.52	0.58	0.18	0.12	2.73E-03
DHFR	EST	Reject	0.52	0.63	0.21	0.13	7.51E-07
	NA	Reject	0.46	0.55	0.13	0.14	5.34E-23
	TK	Reject	0.38	0.51	0.16	0.13	8.03E-11

	DHFR	Pass	0.55	0.62	0.28	0.16	0.10111
	ESA	Reject	0.23	0.48	0.14	0.14	2.29E-31
ESA	EST	Pass	0.67	0.76	0.25	0.14	0.23105
	NA	Reject	0.33	0.59	0.24	0.20	1.51E-58
	TK	Reject	0.46	0.57	0.25	0.20	0.000121

	DHFR	Pass	0.55	0.57	0.21	0.14	4.01E-01
	ESA	Reject	0.25	0.42	0.13	0.12	7.04E-20
EST	EST	Reject	0.31	0.48	0.09	0.12	7.94E-39
	NA	Reject	0.40	0.46	0.15	0.15	1.46E-09
	TK	Reject	0.28	0.43	0.09	0.15	2.17E-29

	DHFR	Reject	0.35	0.68	0.22	0.25	3.46E-25
	ESA	Reject	0.59	0.71	0.28	0.24	2.91E-04
NA	EST	Reject	0.56	0.66	0.25	0.24	2.46E-04
	NA	Reject	0.17	0.73	0.07	0.20	0.00E+00
	TK	Reject	0.48	0.60	0.18	0.23	3.46E-07

	DHFR	Reject	0.42	0.62	0.13	0.10	9.80E-12
	ESA	Reject	0.16	0.52	0.07	0.13	9.99E-62
TK	EST	Pass	0.58	0.65	0.18	0.14	6.28E-02
	NA	Reject	0.40	0.53	0.11	0.15	2.92E-53
	TK	Reject	0.19	0.47	0.08	0.15	3.89E-64

^a^ Standard Deviation

#### Calculating a reference threshold by verifying dataset

Using a verifying dataset, we calculated the distance threshold (correlation coefficient: 0.39) that had the maximum discrimination. The reference threshold of atom-pair (Tanimoto coefficient: 0.55 in Fig. 2) was calculated via 7 classes of structures showing the complement between atom-pair descriptor and protein-ligand interaction descriptor.

### Protein-ligand interaction clustering

#### Cluster analysis of human dihydrofolate reductase molecular docking

**Figure 3:**
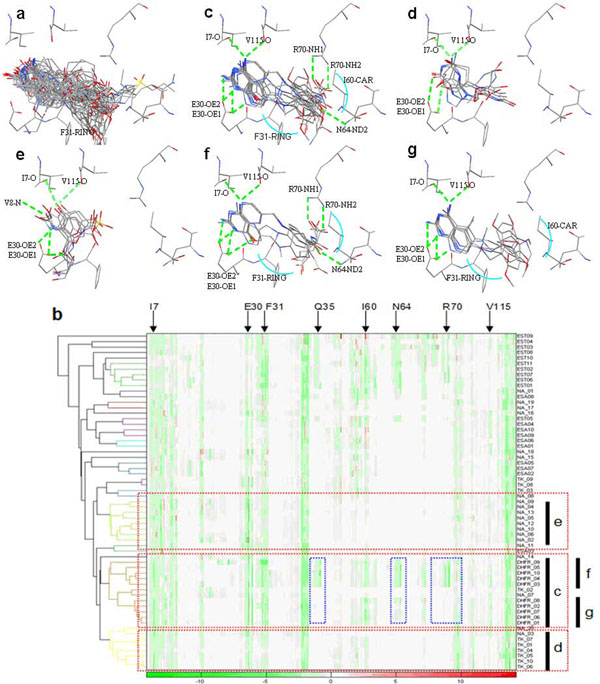
**Cluster analysis of hDHFR**. (a) Overlay of all 61 docked poses of known active compounds in the vicinity of the target protein hDHFR (PDB code: lhfr). (b) The dendrogram and hierarchical clustering results of 61 docked poses of hDHFR. Each cluster has its interaction details in the figures above (e.g. cluster c in fig c). Docked poses in the heat map are rearranged according to the order given by hierarchical clustering marked by the black bar ‘c’ in the right side of the heat map. The amino acids identified for description are shown in the top side of the heat map. (c, d, e) Overlay of the known active compounds and their important interactions, (f, g) Docked poses overlay of the sub-cluster within hDHFR active compounds. The differences of clusters f and g are shown by blue frames in the heat map.

The overlays of all 61 docked poses of known active compounds in the vicinity of the target protein hDHFR are shown in Figure 3a. Using the reference threshold of protein-ligand interaction (correlation coefficient: 0.39), three major clusters can be identified in Figure 3b, clusters c, d and e. Each cluster has interaction details displayed above (e.g. cluster c with fig. c). All active compounds were grouped together (Fig. 3c). The hDHFR ligands in cluster c had hydrogen bonds (E30-OE1, E30-OE2, V115-0, I7-0 in green dotted lines) and van der Waals forces shown by a blue arc (I60-CAR, F31-RING) revealing that binding interactions of each docked pose within cluster c were similar. Cluster d contained 6 TK ligands and one NA ligand and cluster e had only NA ligands, as seen in Figure 3e. Docked poses within both clusters d and e had hydrogen bonding (V115,I7-0; E30-OE1, V8-N).

When comparing the binding interaction between clusters in Figures 3c, d, e, f, and g we noted that our method could cluster docked compound poses into distinct clusters revealing specific binding interactions and important protein-ligand interactions.

#### Cluster analysis of molecular docking on thymidine kinase

After filtering out clustered compounds, 53 docked poses were obtained including the 10 docked poses of active compounds and a total of 305 atoms were identified here. (Fig. S6 in Additional File 7)

### Clustering by atom-pair descriptor

#### Cluster analysis of compound structures for the verifying dataset

**Figure 4:**
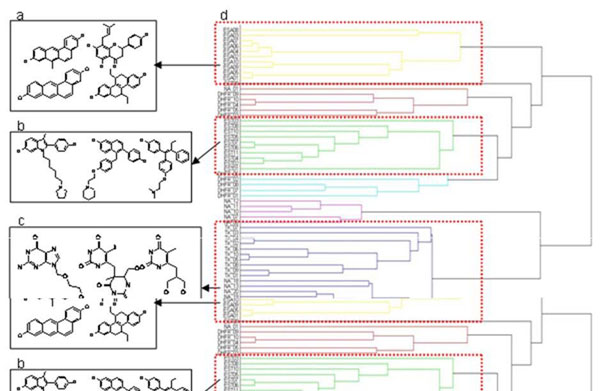
**The dendrogram of hierarchical clustering of 61 known compound structures with three major clusters**. (a) 10 ERα agonists, (b) 11 ERα antagonists, (c) 10 TK and 14 NA inhibitors were grouped into one cluster due to their structure similarity. The descriptor was calculated using the tanimoto coefficient (0.55) via atom-pair representation. It grouped only compounds with similar structures, sorting them out from those with different structures.

Observing these three clusters, we deduced the atom-pair descriptor could group compounds with similar structures and sorts them from those with different structures (Fig. 4).

### Cluster analysis of virtual screening results on the testing dataset

#### Analysis of the hDHFR dataset (first and second stages)

**Figure 5:**
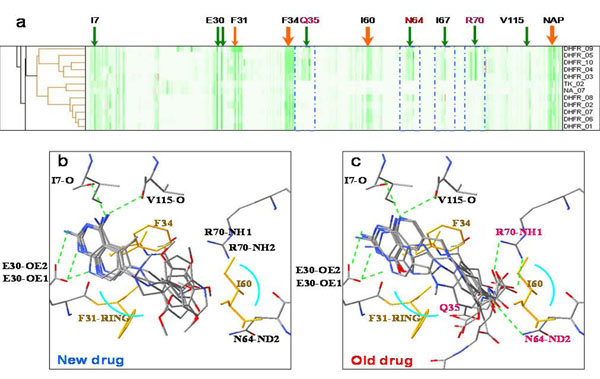
**The detail of hDHFR binding interactions of new drugs and old drugs on the verifying dataset.** (a) Important forces (red arrows) on the heat map (160-van der Waals force, F31-stacking force, F34-stacking force, NAP-stacking force); (b), (c) The binding interactions of new and old drugs and their residues (yellow). The old drug (c) has additional hydrogen bonding with the target protein (Q35, N64, and R70). Interactions of residues (Q35, N64, and R70) are seen in (b) while (N64 and R70) interactions are seen in (c).

**1^st^ stage:** We performed virtual screening for a set of 10 hDHFR inhibitors all spiked into 990 randomly selected compounds from ACD. A total of 476 involved atoms were identified in 100 docked poses that include 10 known active compounds. P-L interactions of all complexes were generated, each complex being composed of 316 real numbers. All hDHFR inhibitors were grouped together into one cluster. In Figure 5a indicated by red arrows are: F31-stacking forces, 160-van der Waals forces and NAP-stacking forces. Figures 5b and 5c shows similar hydrogen bonding (I7-O, V115-O, E30-OE1, E30-OE2, and N64-ND2) for the target protein and the 35 unknown compounds, however, the old drug (Fig. 5c) contains additional hydrogen bonds (R70-NH1, R70-NH2, and N64-ND2). We also identified and pointed out important forces on the heat map using red arrows (I60-van der Waals forces, F31-stacking forces, F34-stacking forces, NAP-stacking forces) Residues within old and new drug structures (Fig. 5a and b) are shown in yellow and the dendrogram in Figure 3b shows the exact split of these two compounds. We utilized 2D topology to select representative compounds within a cluster after protein-ligand interaction analysis was performed and representatives were then selected within each sub-cluster.

**Figure 6:**
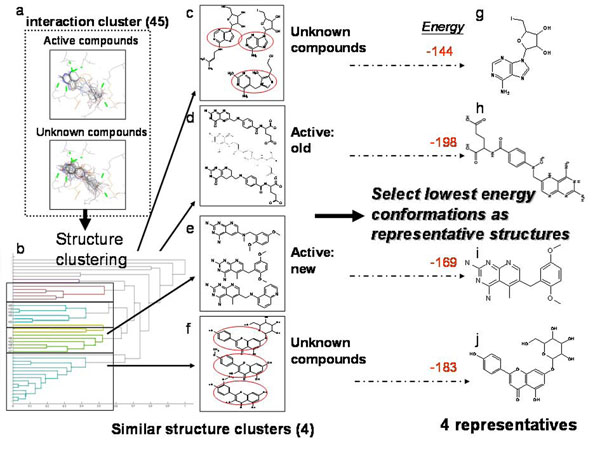
**The process and results of second stage cluster analysis on hDHFR testing dataset.** (a) The binding interactions of the largest cluster generated from first stage clustering: 45 compounds include the 10 active compounds and 35 unknown compounds, (b) The result of hierarchical clustering: there were four major clusters identified by the dendrogram (c, d, e, f). The active compounds were spliced into two clusters: (d) the old drugs and (e) the new drugs due to the difference in carboxylic acid groups. The sub-structures from clusters within the red circles in (c) and (f) had similar compounds and the lowest energy compound within each cluster was selected as a final representative (g), (h), (i) and (j).

**2^nd^ Stage:** The cluster contained 45 compounds: 10 active compounds and 35 unknown compounds (Fig. 6a). A one dimension atom-pair binary string of 2D topology represented each compound. After performing hierarchical clustering four major clusters were identified by the dendrogram (Fig. 6b). The active compounds were spliced into two clusters; the old drugs (Fig. 6d) and the new drugs (Fig. 6e) due to the differences in carboxylic acid groups.The sub-structures within each cluster inside the red circles (Figs. 6c and f) showed similar compounds within a cluster and only the lowest energy compound from each cluster was selected as a final representative (Figs. 6g, h, i and j). At this stage the selected candidates could be further verified by bioassays for specific function and application.

## Discussion

In search of an improved post-screening analysis for protein-ligand complexes we developed a combinatorial cluster analysis aided by two clustering stages to mine and visualize compound candidates generated by VS. Five classes of targets and two different data sets were used to validate this method. In its first clustering stage, our method encodes more interaction-specific information than other methods into the real number string, hydrogen bond, van der Waal and electrostatic forces which are important in receptor-ligand binding increasing the efficiency of protein-ligand interaction clustering. Through second-stage clustering, using physicochemical features as criterion for further screening, final representatives were retrieved from each cluster containing compounds from first-stage clustering.

Another post-screening analysis method VISCANA, uses protein-ligand interactions as a means for clustering but lacks sufficient descriptions of van der Waals forces and hydrogen bond interactions which play an important role in receptor-ligand binding. In addition, its lack of using a specifically optimized docking tool for protein-ligand interactions during virtual screening may undermine the accuracy of final representatives as well.

Our goal was to develop a method for selecting adequate representative compounds from a 3D database that can be used in therapeutic or industrial applications. Such compounds can be further confirmed through bioassays to verify our method's accuracy and the proper activity and application of these final candidates. This study provides a suggestion of cluster threshold while aiding the retrieval of more specific representative structures from a large number of virtual screening data. Furthermore, an overall index criterion to evaluate the accuracy of our clustering method can be done in future studies to enable its comparison with other post-screening analysis methods and thoroughly investigate screening and retrieving advantages and disadvantages of different methods. In future works we hope to extend our TSCC study into the integration or conjunction of our TSCC method with Calibur [6] and NeatMap [3] for the possibility of improving accuracy and specificity in selecting final representatives.

## Conclusions

We showed that by combining interaction clustering with compound structure clustering an enhanced cluster analysis is obtained during the retrieval of final representatives for the five selected targets in this study, simultaneously improving VS enrichment. The overall performance of TSCC revealed that sufficient descriptions of protein-ligand interactions are an important step when mining for ideal protein-ligand complexes. Although comparison to other cluster analysis methods can be somewhat ambiguous since different approaches may vary in goals and purpose, the combination of an optimized docking tool and two clustering stages for the scope of selecting ideal representatives revealed promising results in our study.

## Authors’ contribution

DLC has helped develop, test and implement the steps of this study, organized the entire manuscript. YFC conducted the experiments, analyzed protein docking interfaces and helped to draft the manuscript. CNK developed programs for computing the clustering results and analyzed protein docking interfaces. JMY designed, coordinated and conceived the study, and helped to draft the manuscript. CCL helped with design this study. All authors read and approved the final manuscript.

## Competing interests

The authors declare that they have no competing interests.

## Supplementary Material

Additional File 1 GEMDOCK scoring function. Click here for file

Additional File 2 **Figure S1.** Ten TK (thymidine kinase) active compound structures. Click here for file

Additional File 3 **Figure S2.** Eleven ERα (estrogen receptor) antagonist structures. Click here for file

Additional File 4 **Figure S3.** Ten ERα (estrogen receptor) agonist structures. Click here for file

Additional File 5 **Figure S4.** Ten hDHFR (human dihydrofolate reductase) active compound structures Click here for file

Additional File 6 **Figure S5.** Twenty NA (neuraminidase) active compound structures Click here for file

Additional File 7 **Figure S6.** Views of docked structures of known active compounds in the vicinity of the target protein TK and hierarchical clustering of protein-ligand interactions. Click here for file
