# Supplementary material for: TSCC: Two-Stage Combinatorial Clustering for virtual screening using protein-ligand interactions and physicochemical features
Source: BMC Genomics. 2010 Dec 2;11(Suppl 4):S26. doi: 10.1186/1471-2164-11-S4-S26 (PMC3005922; doi:10.1186/1471-2164-11-S4-S26)
Supplement: Additional File 1 — GEMDOCK scoring function. [file 1471-2164-11-S4-S26-S1.pdf]

GEMDOCK scoring function

The energy function can be dissected into the following terms:

$$E_{tot} = E_{bind} + E_{pharma} + E_{ligpre} \tag{1}$$

where E_{bind} is the empirical binding energy, E_{pharma} is the energy of binding site pharmacophores (hot spots), and E_{ligpre} is a penalty value if a ligand does not satisfy the ligand preferences. E_{pharma} and E_{ligpre} (see Mining pharmacological consensuses subsection) help select active compounds by improving the number of true positives. The values of E_{pharma} and E_{ligpre} are set to zero if active compounds are not available. Thus, the empirical-binding energy (E_{bind}) is given as:

$$E_{bind} = E_{inter} + E_{intra} + E_{penal} \tag{2}$$

where E_{inter} and E_{intra} are the intermolecular and intramolecular energies, respectively, and E_{penal} is a large penalty value if the ligand is out of the range of the search box. For this study, E_{penal} was set to 10,000. The intermolecular energy is defined as:

$$E_{inter} = \sum_{i=1}^{lig} \sum_{j=1}^{pro} \left[F\left(r_{ij}^{B_{ij}}\right) + 332.0 \frac{q_i q_j}{4 r_{ij}^2} \right] \tag{3}$$

where r_{ij} is the distance between the atoms i and j; q_i and q_j are the formal charges and 332.0 is a factor that converts the electrostatic energy into kilocalories per mole. The lig and pro denote the numbers of the heavy atoms in the ligand and receptor, respectively. $F\left(r_{ij}^{B_{ij}}\right)$ is a simple atomic pair-wise potential function as defined in our previous study [5] where $r_{ij}^{B_{ij}}$ is the distance between atoms i and j with interaction type B_{ij} formed by pair-wise heavy atoms between ligands and proteins, B_{ij} is either a hydrogen bond or a steric state. We used the atom formal charge to calculate the electrostatic energy [3], which is set to 5 or −5, respectively. The intramolecular energy of a ligand is:

$$E_{intra} = \sum_{i=1}^{lig} \sum_{j=i+2}^{lig} \left[F\left(r_{ij}^{B_{ij}}\right) + 332.0 \frac{q_i q_j}{4 r_{ij}^2} \right] + \sum_{k=1}^{dihed} A\left[1 - \cos\left(m\theta_k - \theta_0\right)\right] \tag{4}$$

where $F\left(r_{ij}^{B_{ij}}\right)$ is defined as in Equation 3 except the value is set to 1000 when $r_{ij}^{B_{ij}}$

< 2.0 Å, and *dihed* is the number of rotatable bonds in a ligand. We followed the work of Gehlhaar et al. [] to set the values of A, m, and θ_0. For the sp^3-sp^3 bond, $A = 3.0$, $m = 3$, and $\theta_0 = \pi$; for the sp^3-sp^2 bond, $A = 1.5$, $m = 6$, and $\theta_0 = 0$.

When known active ligands were available, GEMDOCK used a pharmacophore-based scoring function (Equation 1). If known active compounds were not available LP_{elec} and LP_{hb} were set to zero and GEMDOCK used a purely empirical-based scoring function (Equation 2). After all of the protein-ligand interactions were calculated, the atom interaction-profile weight of the target protein representing the pharmacological consensus of a particular interaction was given as:

$$Q_j^k = \frac{f_j^k}{N} \qquad (5)$$

where N is the number of known active compounds and f_j^k is the total interaction number of an atom j (in a protein) interacting with an atom of known active ligands with the interaction type k (e.g., hydrogen bonding or hydrogen-charged interactions). An atom j in the reference protein was considered a hot-spot atom when Q_j^k was more than 0.5.
