# Supplementary figures and images for: TSCC: Two-Stage Combinatorial Clustering for virtual screening using protein-ligand interactions and physicochemical features
Source: BMC Genomics. 2010 Dec 2;11(Suppl 4):S26. doi: 10.1186/1471-2164-11-S4-S26 (PMC3005922; doi:10.1186/1471-2164-11-S4-S26)

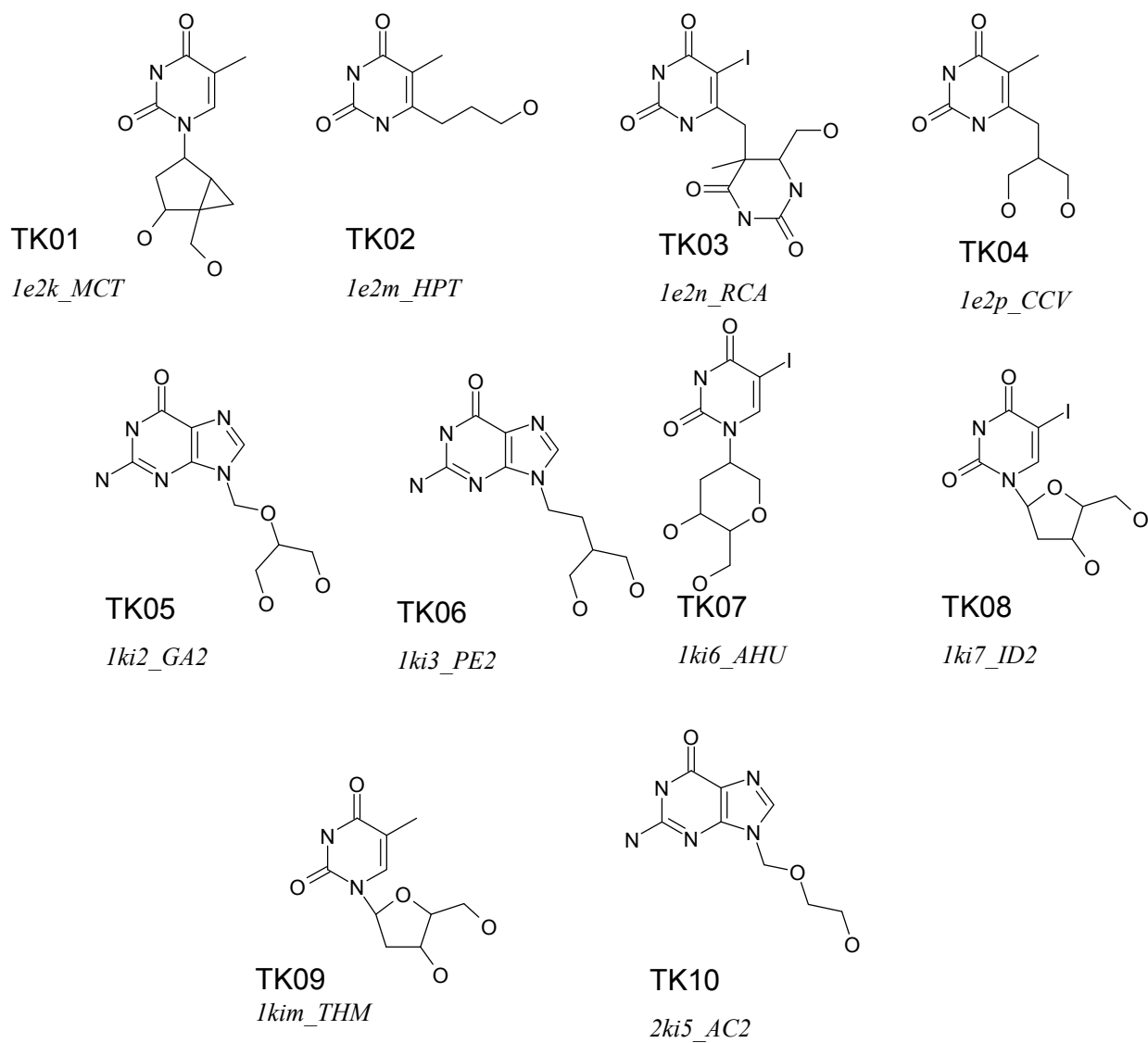

**Figure S1.** Ten TK (thymidine kinase) active compound structures.

Supplement: Additional File 2 — Figure S1. Ten TK (thymidine kinase) active compound structures. [file 1471-2164-11-S4-S26-S2.pdf]

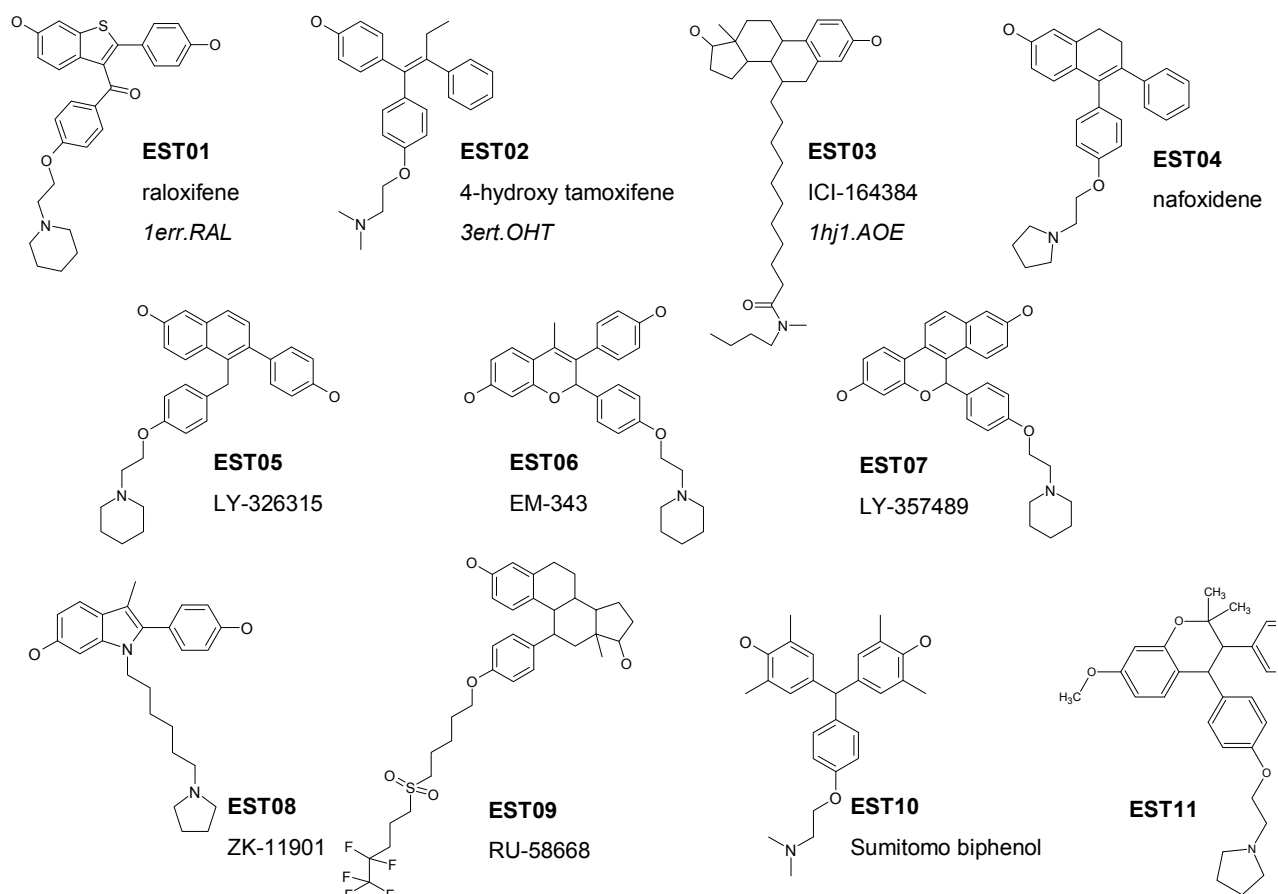

**Figure S2.** Eleven ER $\alpha$  (estrogen receptor) antagonist structures.

Supplement: Additional File 3 — Figure S2. Eleven ERα (estrogen receptor) antagonist structures. [file 1471-2164-11-S4-S26-S3.pdf]

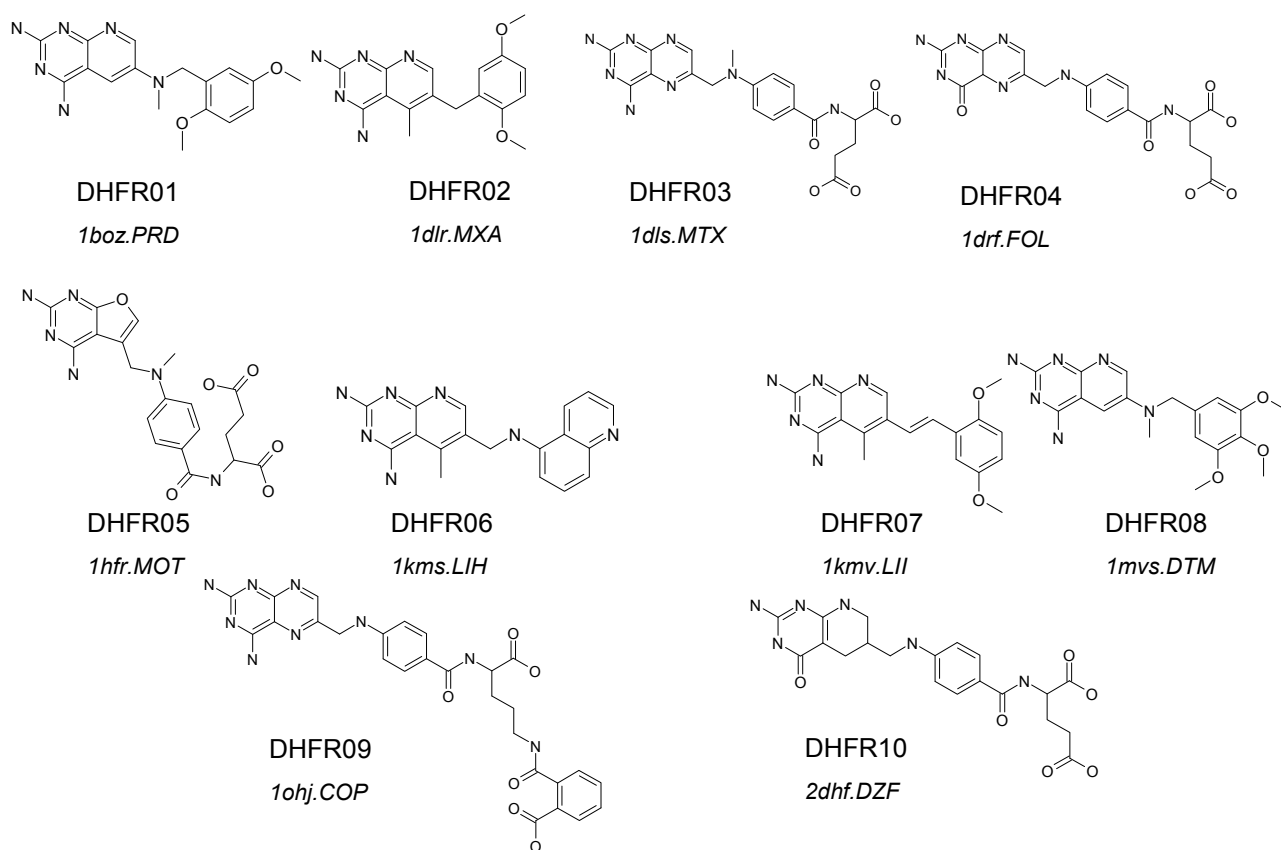

**Figure S4.** Ten hDHFR (human dihydrofolate reductase) active compound structures

Supplement: Additional File 5 — Figure S4. Ten hDHFR (human dihydrofolate reductase) active compound structures [file 1471-2164-11-S4-S26-S5.pdf]
