# Supplementary material for: TSCC: Two-Stage Combinatorial Clustering for virtual screening using protein-ligand interactions and physicochemical features
Source: BMC Genomics. 2010 Dec 2;11(Suppl 4):S26. doi: 10.1186/1471-2164-11-S4-S26 (PMC3005922; doi:10.1186/1471-2164-11-S4-S26)
Supplement: Additional File 4 — Figure S3. Ten ERα (estrogen receptor) agonist structures. [file 1471-2164-11-S4-S26-S4.pdf]

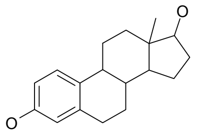

ESA01

estradiol

1gwr_EST

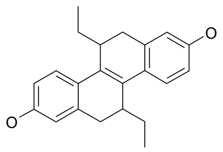

ESA02

(R,R)-5,11-cis-diethyl-5,6,11,12-tetrahydrochrysene-2,8-diol

1l2i_ETC

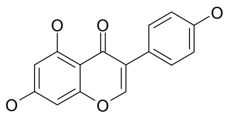

ESA03

genistein

1qkm_GEN

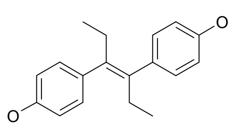

ESA04

diethylstilbestrol

3erd_DES

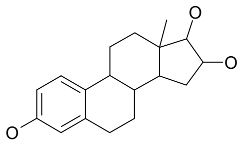

ESA05

estriol

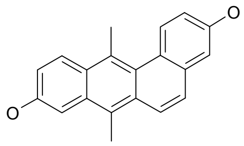

ESA06

7,12-dimethylbenz[a]anthracene-3,9-diol

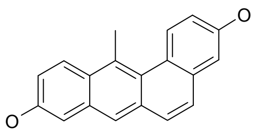

ESA07

12-methylbenz[a]anthracene-3,9-diol

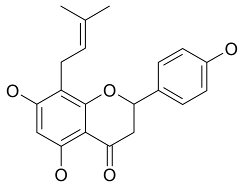

ESA08

8-prenylnaringenin

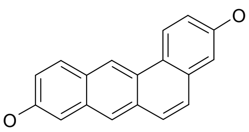

ESA09

benz[a]anthracene-3,9-diol

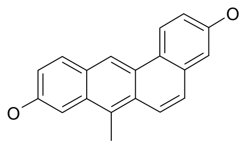

ESA10

7-methylbenz[a]anthracene-3,9-diol

Figure S3. Ten ERα (estrogen receptor) agonist structures.
