# Supplementary material for: TSCC: Two-Stage Combinatorial Clustering for virtual screening using protein-ligand interactions and physicochemical features
Source: BMC Genomics. 2010 Dec 2;11(Suppl 4):S26. doi: 10.1186/1471-2164-11-S4-S26 (PMC3005922; doi:10.1186/1471-2164-11-S4-S26)
Supplement: Additional File 6 — Figure S5. Twenty NA (neuraminidase) active compound structures [file 1471-2164-11-S4-S26-S6.doc]

**Figure S5**. Twenty NA (neuraminidase) active compound structures
