# Supplementary material for: TSCC: Two-Stage Combinatorial Clustering for virtual screening using protein-ligand interactions and physicochemical features
Source: BMC Genomics. 2010 Dec 2;11(Suppl 4):S26. doi: 10.1186/1471-2164-11-S4-S26 (PMC3005922; doi:10.1186/1471-2164-11-S4-S26)
Supplement: Additional File 7 — Figure S6. Views of docked structures of known active compounds in the vicinity of the target protein TK and hierarchical clustering of protein-ligand interactions. [file 1471-2164-11-S4-S26-S7.pdf]

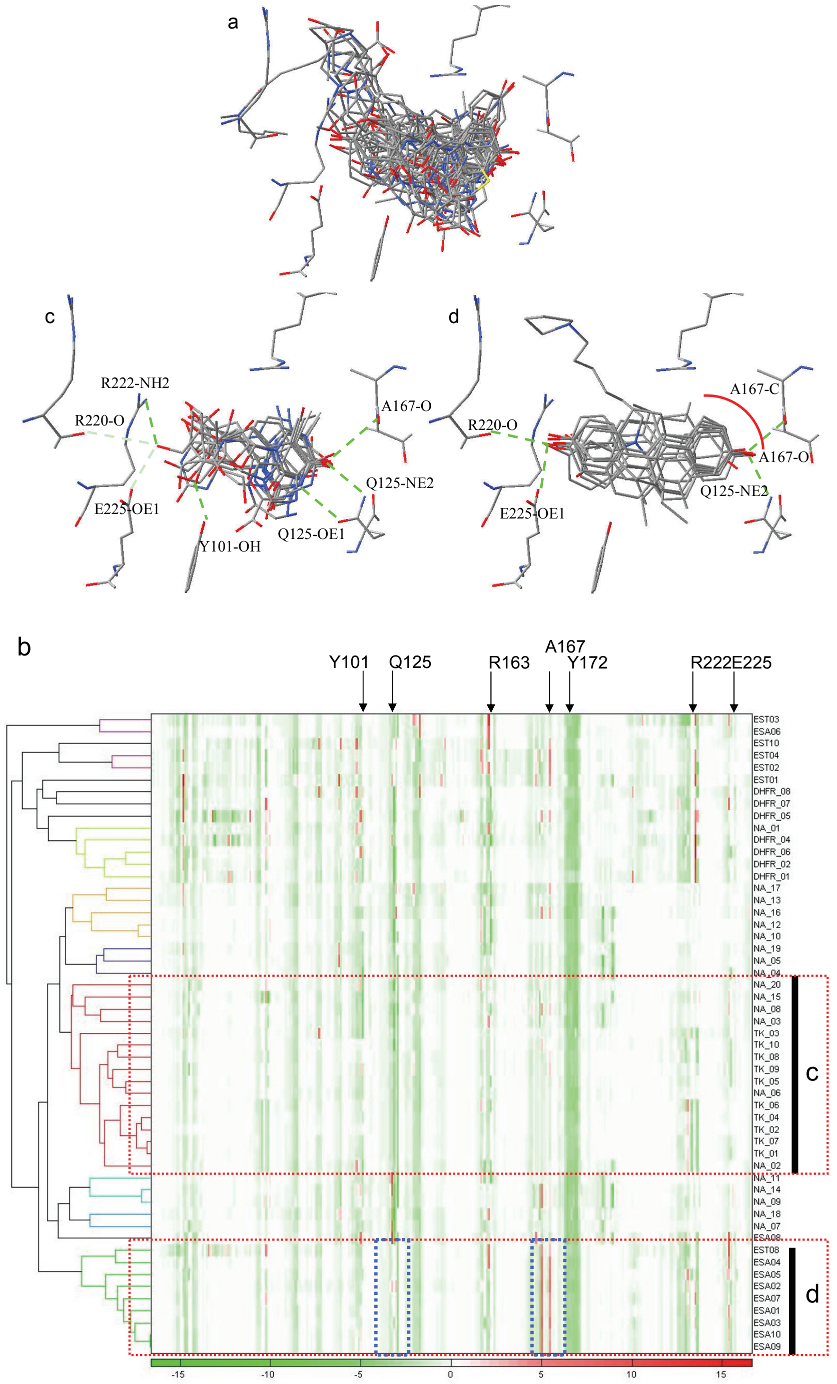

Figure S6. **Views of docked structures of known active compounds in the vicinity of the target protein TK and hierarchical clustering of protein-ligand interactions.**
(a) Overlay of all 53 docked poses of known active compounds in the vicinity of the target protein TK (PDB id: 1kim). (b) Hierarchical clustering of protein-ligand interaction of 53 docked poses on TK (PDB id: 1kim). Each docked pose is represented as one line in the heat map in the middle of the figure, the red being the lowest protein-ligand interaction energy and the green being the highest energy. The left side of the heat map shows the hierarchical clustering results of TK, including the dendrogram. Docked poses in the heat map are rearranged according to the order given by hierarchical clustering marked by the black bar 'c' in the right side of the heat map. The hot spots identified from known overlapping active compounds were also shown at the top side of the heat map. (c) Overlay of docked poses of the cluster with most number of known active compounds and important hydrogen bonds between protein and ligand. (d) Overlay of docked poses of the cluster with most number of unknown compounds and important hydrogen bonds between protein and ligand. The blue frames in the heat map were the major interaction that differ cluster c and d.
